# Gold-decorated vertically aligned carbon nanofibers for high-performance room-temperature ethanol sensing

**DOI:** 10.1007/s00604-025-07367-8

**Published:** 2025-07-24

**Authors:** Mostafa Shooshtari

**Affiliations:** 1 Neuromorphic Group, Institute of Microelectronics of Seville IMSE-CNM, 28. Parque Científico y Tecnológico Cartuja, Sevilla, 41092 Spain; 2 Department of Microelectronics, Delft University of Technology, Feldmannweg 17, Delft, 2628 CT the Netherlands

**Keywords:** Carbon nanofibers (CNFs), Gas sensing, Gold nanoparticles (Au NPs), Volatile organic compounds (VOCs), Plasma-enhanced chemical vapor deposition (PECVD), Aerosol jet printing, Room-temperature sensors

## Abstract

Room-temperature detection of volatile organic compounds (VOCs) is a persistent challenge for low-power sensing applications. This study presents a scalable method for fabricating gas sensors based on vertically aligned carbon nanofibers (VACNFs) decorated with aerosol-printed gold nanoparticles (Au NPs). The Au-functionalized CNFs show ~ 140% improvement in response at 10 ppm ethanol gas, reduced ~ 5-s response time, and enhanced stability with over 95% signal retention. These improvements are attributed to increased surface area, enhanced graphitic disorder, and the catalytic activity of Au NPs. Structural and morphological characterization using TEM, EDX, SEM, and Raman spectroscopy confirms the successful decoration of CNFs with Au. The effects of operating temperature and humidity on sensor performance are also evaluated.

**Graphical Abstract:**

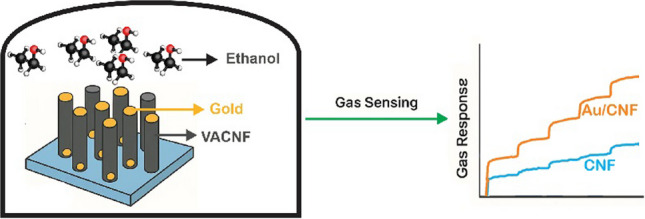

**Supplementary Information:**

The online version contains supplementary material available at 10.1007/s00604-025-07367-8.

## Introduction

Carbon nanofibers (CNFs) have emerged as versatile materials for gas sensing, thanks to their unique structural and electronic properties [1]. Since their discovery, CNFs have been widely studied for applications in various sensing platforms due to their high electronic conductivity and large surface-to-volume ratio, both of which are essential for achieving high sensitivity in gas sensors [2]. In addition to these inherent advantages, CNFs demonstrate a remarkable ability to adsorb gas molecules on their surface, allowing them to detect changes in the surrounding environment with high specificity [3–6]. Compared to other carbon-based nanostructures, CNFs are particularly promising due to their one-dimensional (1D) morphology, which enhances charge transport along the fiber axis and improves response time for dynamic sensing applications [7, 8].

One approach to optimize CNF-based gas sensors is through the use of vertically aligned CNF arrays produced by chemical vapor deposition (CVD) [9–11]. This fabrication technique enables precise control over CNF properties, such as diameter, length, and density, which are crucial parameters influencing sensor performance. By adjusting CVD conditions, such as temperature, precursor concentration, and catalyst composition, researchers can fine-tune these properties to maximize surface area and electronic interactions with analytes [12]. Controlling CNF density, in particular, allows for tailored sensor architectures where the number of fibers per unit area can directly affect gas molecule adsorption and charge transfer processes. While other carbon nanomaterials, like carbon nanotubes (CNTs), offer similar functionalities, achieving uniform alignment and density control is often more feasible in CNF arrays.

According to these advantages, most research to date has focused on enhancing CNF synthesis methods and exploring gas sensing mechanisms. Metal nanoparticles (NPs) have also attracted attention in recent years as functional enhancements for CNF-based gas sensors [13, 14]. Owing to their distinct electrical, optical, and catalytic properties, metal NPs, especially those composed of noble metals like gold [15], silver [16], and platinum, have been integrated into CNF frameworks to improve sensor response. Decorating CNFs with metal NPs introduces new charge transport pathways and catalytic sites that facilitate stronger interactions with gas molecules [17]. For instance, metal NPs can serve as electron donors or acceptors, creating localized charge carrier modulation that amplifies the signal generated upon gas exposure. Additionally, metal NPs are highly reactive toward certain gas species, enabling selective detection of analytes such as ammonia, hydrogen sulfide, and carbon monoxide, which are of particular interest in public health and industrial safety. Recent studies have further demonstrated that noble metal decoration can lead to the formation of heterojunctions, which enhance gas sensing performance through synergistic effects such as band alignment modulation and interfacial charge redistribution [18, 19].

Recent studies on carbon family materials show that noble metal NPs, such as gold (Au), can significantly enhance the selectivity and sensitivity of CNFs to various gas analytes [17, 20, 21]. When Au NPs are deposited on CNFs, they contribute free electrons to the CNF matrix, promoting stronger gas-CNF interactions and thereby boosting the sensor’s response. In particular, studies have demonstrated that Au-decorated CNF composites show promising potential in detecting VOCs, which is of interest for applications ranging from environmental monitoring to breath analysis for medical diagnostics.

Volatile organic compounds (VOCs) are a class of organic chemicals that easily vaporize at room temperature (RT) and are found in various natural and industrial environments. As there is rapid growth in the development of environmental sensors, such as gas sensors [22–25] or humidity sensors [26, 27], monitoring VOCs is critically important due to their direct impact on environmental and human health. Many VOCs, such as benzene, formaldehyde, and toluene, are classified as hazardous air pollutants that contribute to air quality degradation and have been linked to respiratory illnesses, cancer, and neurological disorders. In indoor environments, VOCs are emitted from common household products like paints, adhesives, and cleaning agents, posing potential long-term health risks with prolonged exposure. In industrial settings, VOCs are byproducts of processes such as manufacturing, petrochemical refining, and waste treatment. These compounds can contribute to air pollution by reacting with nitrogen oxides in the atmosphere to produce ground-level ozone and secondary organic aerosols, both of which exacerbate urban smog and respiratory issues. Beyond environmental and health concerns, VOC monitoring is increasingly valuable for medical diagnostics, as certain VOCs in exhaled breath can serve as biomarkers for diseases such as diabetes, lung cancer, and infections. Thus, the ability to accurately detect and quantify VOCs is essential for regulatory compliance, public health protection, and the development of non-invasive diagnostic tools, highlighting the growing demand for highly sensitive and selective VOC sensors [28, 29].

In this work, I present a study on CNFs synthesized via PECVD, which were then decorated with Au NPs by printing technique to investigate their efficacy as VOC vapor gas sensors. My study examines the role of gold nanoparticles as a key parameter in sensor performance. I analyze how gold decoration influences gas sensing properties, especially at room temperature, over a concentration range of 1–20 ppm ethanol, providing insights that could guide future developments in CNF-based gas sensor design. My findings are supported by transmission electron microscopy (TEM) analysis, scanning electron microscopy (SEM), and Raman spectroscopy, along with gas response measurements, which together offer a detailed perspective on the relationship between CNF density, nanoparticle decoration, and sensing efficiency. The Au/CNF sensors demonstrated high improvement in gas response and significantly faster response time compared to pristine CNFs. These results contribute to the broader understanding of how CNF-based materials can be configured for optimal sensitivity, selectivity, and stability in gas sensing applications.

## Experimental

### Materials growth

Aligned bundles of carbon nanofibers (CNFs) were synthesized on a silicon wafer with a 9-nm patterned nickel catalyst layer using plasma-enhanced chemical vapor deposition (PECVD). The deposition was performed in an AIXTRON Blackmagic CVD reactor, following the parameters outlined in [30]. The growth process took place at a pressure of approximately 9 mbar, with 700 sccm of hydrogen (H₂) introduced into the chamber while the temperature was gradually raised to 500 °C. Once the target temperature was reached, acetylene (C₂H₂) gas was added at varying flow rates between 5 and 25 sccm to control the CNF density. Growth proceeded for 20 min under a 100 W pulsed-DC plasma, after which the reactor was cooled under a nitrogen (N₂) atmosphere to complete the process.

To decorate the CNF samples with gold nanoparticles (Au NPs), I employed an aerosol-based nanoparticle printing technique, which is well-suited for producing high-purity nanostructured materials with precise control over particle deposition [31]. In this study, gold nanoparticles were generated using a VSP G1 spark discharge generator (VS Particle B.V.), equipped with 99.999% pure Au electrodes and utilizing nitrogen (N₂) gas as the carrier at a flow rate of 1.5 L/min. The generator operated at 1 kV and 8 mA, producing a stable aerosol that was directed to a custom nanomaterial printer prototype developed by VS Particle B.V. This system integrates a programmable xyz-stage and an inertial impactor to enable precise, direct-write deposition of nanoparticles. The aerosol printer was set to operate at a low pressure (< 1 mbar) and ambient temperature, creating nanoporous deposits with a high surface area, which are advantageous for sensor and catalytic applications. The deposition characteristics, such as line or dot size, were controlled by adjusting the nozzle distance, writing speed, and deposition time. Additionally, nanoparticle size could be modified by altering the generator parameters. For this experiment, Au nanostructures were printed at three distinct locations on each sample, with a spacing of 2 mm between deposition points. Using a nozzle with a 0.3 mm orifice, the nozzle was positioned 0.5 mm above the sample surface in a chamber maintained at 0.7 mbar, and each dot deposition was carried out for approximately 5 s.

### Measurement setup

**Fig. 1:**
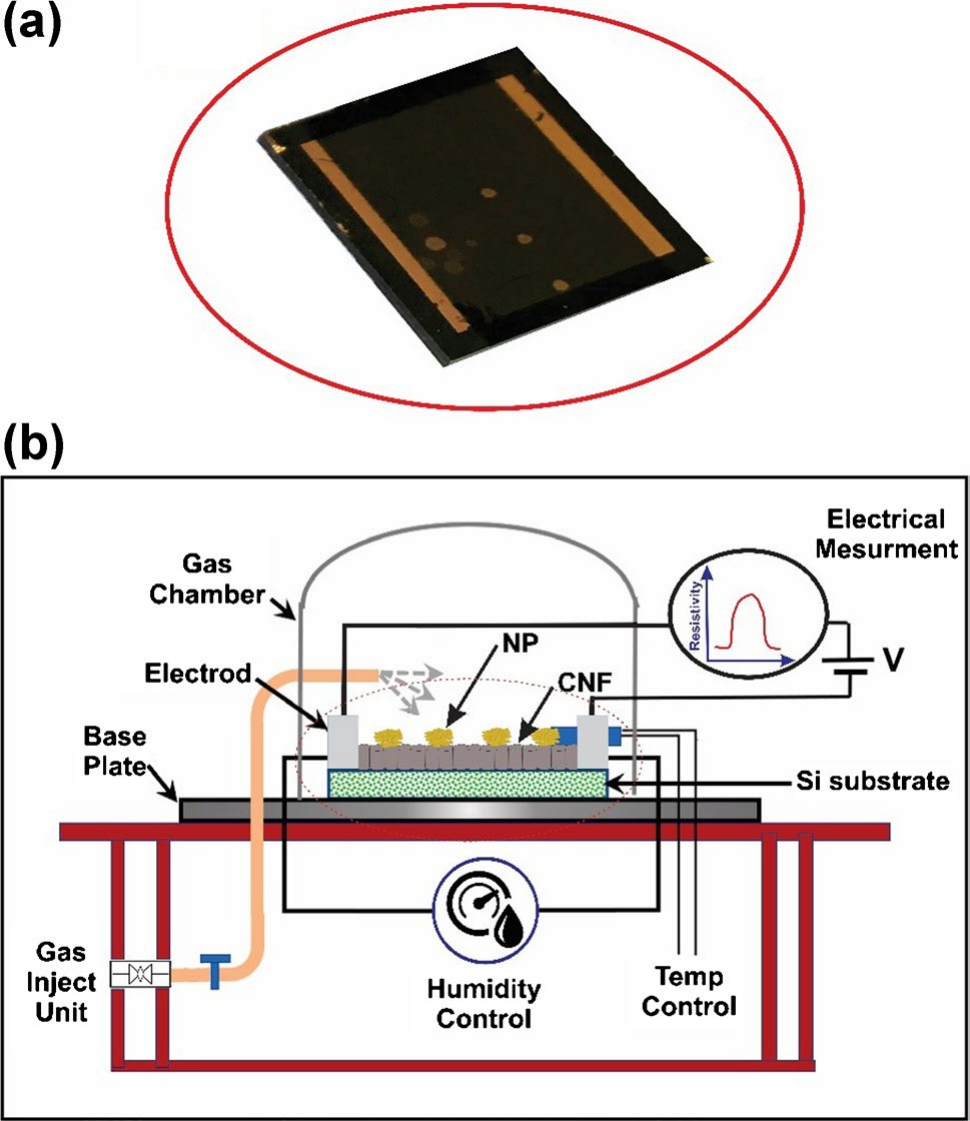
**a** Image of the final fabricated gas sensor sample (1 cm × 1 cm), cut from a 4″ wafer and equipped with 100 nm-thick parallel gold electrodes (with a 10 nm Ti adhesion layer) deposited via e-beam evaporation through a shadow mask. **b** Experimental setup showing the sensor placed inside a controlled test chamber connected to external measurement instrumentation (Keithley 2600B SMU), used to evaluate sensor response to ethanol vapors under regulated temperature and humidity conditions

Square samples, each measuring 1 cm × 1 cm and cut from a 4″ wafer, were prepared for gas sensing tests. The final fabricated sample is shown in Fig. 1a. To facilitate sensing, 100 nm gold electrodes (or platinum) were applied to the sample surface using an e-beam evaporator with a shadow mask. These electrodes, 1 mm in width, were positioned as two parallel strips at the sample edges, with a gap of approximately 8 mm between them serving as the active sensing region. For stable adhesion to the silicon oxide surface, a 10-nm titanium layer was used under gold electrodes, and a 10-nm tantalum layer was used under platinum electrodes. Although both electrode types were tested, final results are reported based on samples with gold electrodes to assess the impact of electrode material on sensor performance.

During testing, the samples were placed in a chamber with controlled analyte concentration, humidity, and temperature, monitored by an ACS Discovery E-series system. Electrical connections from the electrodes to external wiring were established with conductive carbon paste. Sensor response was quantified as follows:

1 $$\text{SR}=\frac{{R}_{\text{g}}-{R}_{\text{a}}}{{R}_{\text{a}}} \times 100\%$$

where *R*_g_ and *R*_a_ represent the sensor resistance after exposure to the analyte and in ambient conditions (25 °C and 55% relative humidity), respectively. Measurements were conducted using a Keithley 2600B Source Measure Unit with a 1 V DC bias. Sensor responses were recorded at varying ethanol vapor concentrations, introduced by evaporating precise amounts of liquid ethanol measured with a microsampler. Ethanol concentration in the chamber was determined by the mass ratio of ethanol to air. This gas sensing protocol follows the methodology established in prior studies [32–34]. Figure 1b illustrates the setup, showing the fabricated sensor within the chamber and the external electronic measuring equipment.

Ethanol vapor with specific concentrations was generated by injecting precise volumes of high-purity liquid ethanol (Merck/1.00983.1000) into a sealed 5-L glass chamber. The volume of liquid ethanol was measured using a calibrated micro-sampler. After injection, the liquid was allowed to fully evaporate, and the resulting vapor was assumed to mix homogeneously with the air in the chamber. Ethanol concentration was estimated based on the injected volume and calculated using the ideal gas law. This method ensured reliable and reproducible gas-phase concentration control for sensing measurements.

## Results and discussion

### Characterization

**Fig. 2:**
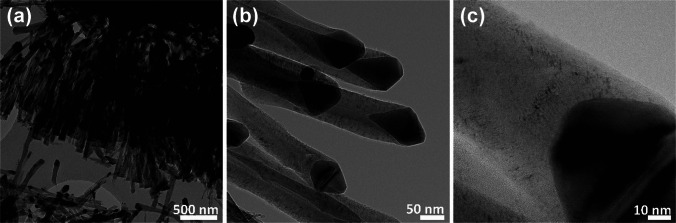
TEM images of a single vertically aligned carbon nanofiber (VACNF) synthesized via DC-PECVD at different magnifications: **a** low-magnification overview (scale bar, 500 nm), **b** mid-magnification view highlighting the herringbone-like graphitic structure (scale bar, 50 nm), and **c** high-magnification image of the CNF tip with a metallic nanoparticle (scale bar, 10 nm), confirming a tip-growth mechanism. The graphitic stacking and exposed edge planes enhance surface reactivity for gas sensing and Au NP functionalization

To achieve reproducible growth of vertically aligned CNFs (VACNFs) with uniform morphology, critical synthesis parameters, including catalyst thickness, growth temperature, plasma power, chamber pressure, and deposition time, were carefully optimized and kept constant across all samples. Morphological inspection and structural characterization of the individual CNFs were conducted using a high-resolution transmission electron microscope (TEM: FEI-Tecnai, G2 200 kV), allowing detailed insight into nanoscale features that directly influence sensing performance**.** Figure 2 presents a representative TEM micrograph of a single CNF, revealing a well-defined cylindrical structure with a disordered graphitic architecture. The CNFs exhibit a herringbone-like stacking of graphitic planes, which is a hallmark of carbon nanofibers synthesized by PECVD. This stacking geometry creates a high density of exposed edge-plane sites, which are chemically more active and hence beneficial for gas adsorption and surface functionalization [35].

The average diameter of the CNFs was determined to be approximately 50 ± 5 nm, consistent with prior reports on nickel-catalyzed CNF growth under similar PECVD conditions [11, 36]. The uniformity in fiber diameter across multiple samples suggests high process reproducibility. Furthermore, the presence of a metal nanoparticle at the tip of the CNF, as shown in the inset of Fig. 2, confirms a tip-growth mechanism, a growth mode commonly observed when the catalyst remains weakly bound to the substrate during thermal decomposition of hydrocarbon feedstock [37]. This growth mechanism ensures that the catalyst remains active throughout the growth process, facilitating the formation of vertically aligned nanostructures. This graphitic arrangement, combined with the narrow diameter distribution, is crucial for sensing applications, as it maximizes the accessible surface area while preserving mechanical robustness. Additionally, the disordered graphitic shells offer pathways for enhanced electronic interaction with adsorbed analyte molecules, especially when functionalized with noble metal nanoparticles.

SEM is an essential tool for analyzing both the structure and composition of CNF-based surface layers, which are key for gas sensing applications. The morphology of CNFs critically influences their sensing capability by increasing available surface area for adsorption and creating favorable electronic properties. In this study, I used a Hitachi Regulus-8230 SEM to examine the microstructures of the CNF samples.

**Fig. 3:**
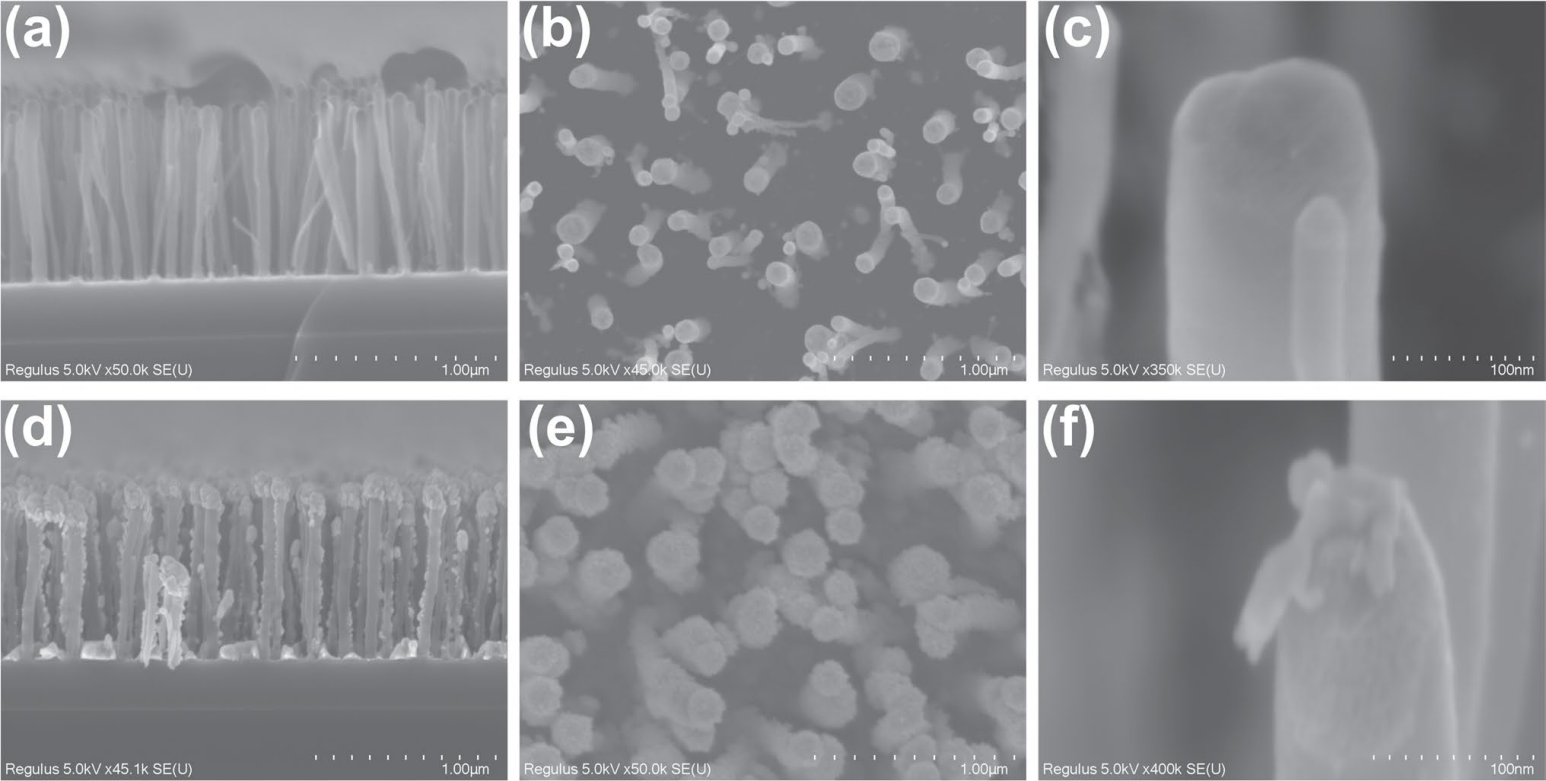
SEM micrographs of carbon nanofibers (CNFs) synthesized via DC-PECVD using a 6-nm nickel catalyst at 600 °C. **a** Cross-sectional view illustrating vertically aligned CNFs with lengths of approximately 1.5 µm and diameters between 50 and 100 nm. **b** Top-view image showing the effect of catalyst patterning on CNF growth morphology. **c** Higher-magnification views emphasizing fiber uniformity. **d**–**f** SEM images of CNFs decorated with gold nanoparticles (NPs) deposited via aerosol impaction printing, revealing sub-10 nm NPs concentrated on fiber tips due to top-down deposition, aimed at enhancing gas sensor sensitivity

Figure 3a–c provide SEM images of CNFs synthesized via DC-PECVD, using a 6-nm nickel catalyst layer at 600 °C. Figure 3a shows a cross-sectional view image that demonstrates the uniform alignment of fibers and allows length estimation, while Fig. 3b offers a top-view, highlighting the role of catalyst patterning on fiber growth. The images reveal well-ordered fibers, with diameters ranging from 50 to 100 nm and lengths around 1.5 µm. This diameter range is consistent with previously reported CNF synthesis results [11] and is slightly larger than typical CNTs used in gas sensors. This slight increase in diameter could improve mechanical stability but may also reduce the specific surface area, potentially affecting the gas adsorption efficiency [38].

To enhance sensor sensitivity, gold nanoparticles (NPs) were deposited onto the CNF surface using aerosol impaction printing. SEM micrographs, shown in Fig. 3d–f, confirm the presence of these nanostructures on the fibers’ surfaces. As seen in Fig. 3d–f, the NPs are primarily concentrated on the tops of the fibers, reflecting the top-down particle flow during deposition. The NPs have an estimated diameter of less than 10 nm, consistent with dimensions reported in similar studies on spark-ablated NPs [39, 40].

**Fig. 4:**
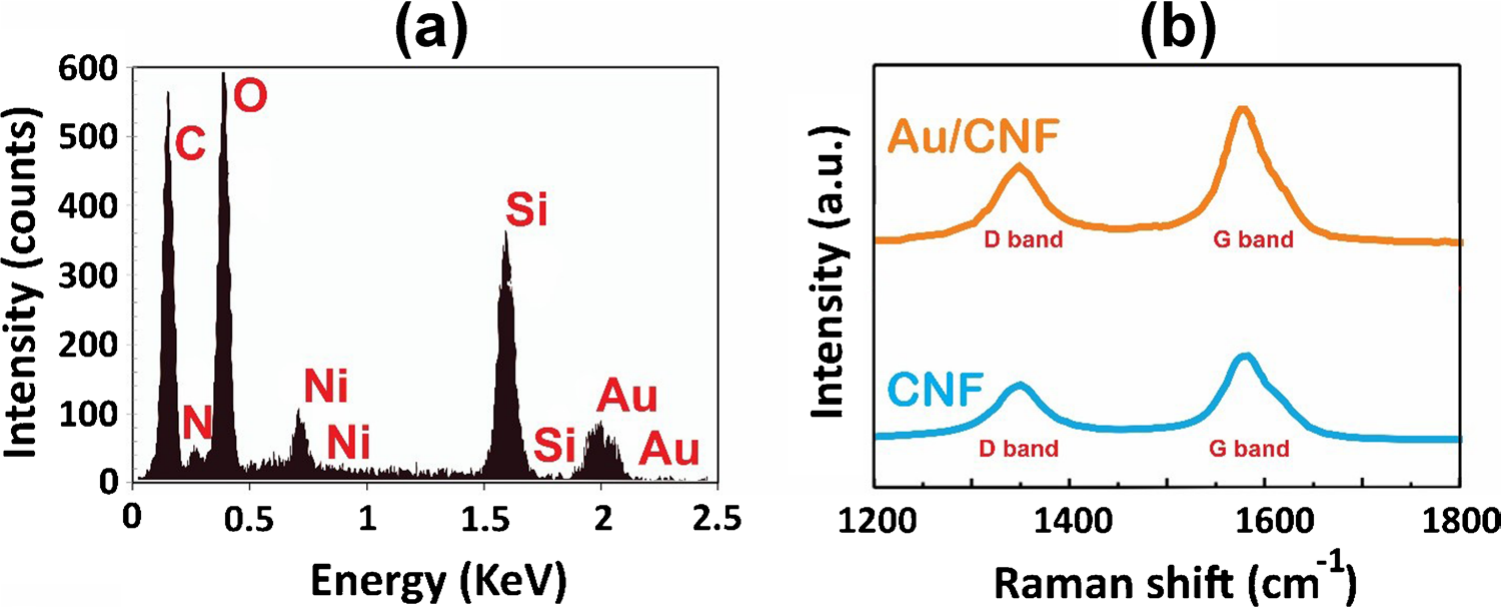
**a** EDX spectrum of Au-decorated carbon nanofibers (CNFs), confirming the presence of gold (Au) alongside carbon (C), silicon (Si), oxygen (O), and nickel (Ni), with no detectable contamination. Au peaks verify successful nanoparticle deposition, while Si and O originate from the substrate and Ni from the growth catalyst. **b** Raman spectra of bare and Au-decorated CNFs showing characteristic D and G bands. The increased *I*_D_*/I*_G_ ratio from 0.25 to 0.39 and the G-band shift from 1579 to 1596 cm⁻^1^ after decoration indicate induced structural disorder and charge transfer between Au NPs and the CNF matrix, relevant to enhanced sensing performance

Also, to confirm the elemental composition and investigate the structural modifications induced by gold nanoparticle (Au NP) decoration, energy dispersive X-ray spectroscopy (EDX: FEI-Nova-NanoSEM 450, EDAX Octane detection) and Raman spectroscopy (Renishaw-inVia) were performed. The corresponding results are presented in Fig. 4.

The EDX spectrum of the decorated CNF sample, shown in Fig. 4a, confirms the successful incorporation of Au onto the CNF surface. Distinct Au peaks are observed alongside those of C, Si, O, and Ni (EDS quantitative elemental composition is presented in Table S1). The Si and O signals originate from the underlying silicon oxide substrate, while the Ni peak stems from the residual catalyst layer used during CNF growth. Importantly, the absence of other metallic signals verifies the chemical purity of the deposited nanoparticles and the cleanliness of the fabrication process.

The detection of Au signals, particularly at the characteristic Lα (9.71 keV) and Mα (2.12 keV) lines, affirms the presence of metallic Au, which plays a critical role in enhancing gas sensing. Au NPs introduce high surface energy sites that facilitate the adsorption of volatile organic compounds (VOCs), and they can serve as active sites for charge transfer, thereby modulating the local carrier density in the CNFs [41]. In noble metal–decorated carbon-based sensors, such as this system, the Schottky junction formed at the metal–carbon interface promotes electron transfer upon gas adsorption, leading to measurable changes in resistance [42].

Raman spectroscopy was employed to evaluate structural quality and probe the interaction between Au NPs and the CNF surface. As shown in Fig. 4b, both pristine and decorated samples exhibit the typical D-band (~ 1350 cm⁻^1^), associated with disordered carbon or edge defects, and the G-band (~ 1579–1596 cm⁻^1^), characteristic of in-plane vibrations of sp^2^-bonded carbon atoms. Following decoration, the intensity ratio *I*_D_/*I*_G_ increased from 0.25 to 0.39, indicating a higher degree of structural disorder or surface modification. This increase is not necessarily detrimental; in fact, moderate defect introduction can enhance gas adsorption by increasing the number of active sites for molecular binding [43]. Furthermore, the observed G-band upshift from 1579 to 1596 cm⁻^1^ suggests phonon stiffening, likely resulting from charge transfer between Au and the π-electron system of the CNFs. Similar spectral shifts have been reported in noble-metal-decorated graphitic nanomaterials and are attributed to hybridization effects and localized surface plasmon interactions [44].

These Raman findings support the hypothesis that Au NPs do not merely sit passively on the CNF surface but actively interact at the electronic level, altering the local density of states and enabling improved sensitivity to ambient VOC molecules. This chemical interaction can also influence the sensor’s selectivity profile, as the Au surface can catalyze specific oxidation reactions depending on the analyte’s redox properties [45]. Overall, the combined EDX and Raman analyses confirm not only the presence of Au but also the structural and electronic modifications imparted to the CNF matrix, both of which are essential for the enhanced gas sensing performance demonstrated later in this study.

### Gas sensing performance

The gas sensing characteristics of the CNF-based sensors were evaluated by monitoring the change in electrical resistance in response to different concentrations of ethanol vapor at room temperature. Both pristine CNFs and Au-decorated CNFs (Au/CNF) were tested under identical conditions to investigate the impact of Au nanoparticle (NP) functionalization on sensing behavior.

**Fig. 5:**
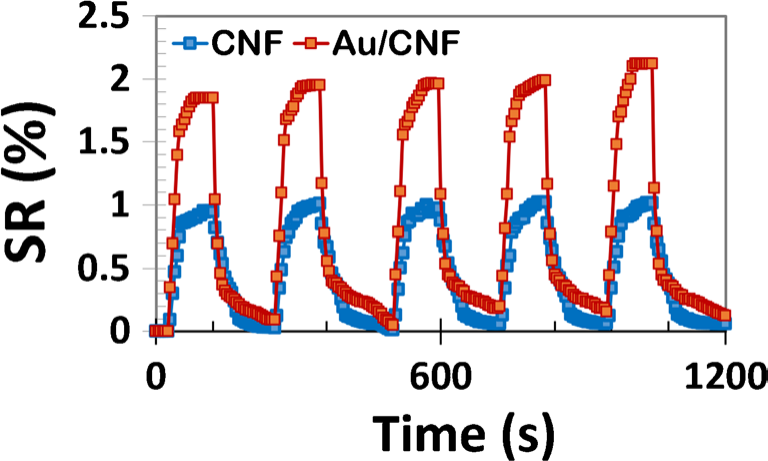
Five adsorption/desorption cycles for CNF and Au/CNF exposed to 1 ppm vapors of ethanol at room temperature

To further investigate the dynamic sensing behavior, the real-time response and recovery profiles of both bare CNF and Au/CNF sensors upon exposure to 1 ppm ethanol vapor were recorded at room temperature, as shown in Fig. 5. Both sensors exhibit a characteristic time-dependent increase in resistance upon ethanol exposure, followed by a gradual recovery once the analyte is purged from the chamber. However, a significant improvement is observed in the Au/CNF sensor. Not only does the Au/CNF structure reach its peak response faster, but it also recovers more effectively within the same measurement window. In contrast, the CNF sensor shows slower adsorption kinetics and fails to fully return to its baseline resistance, indicating poor reversibility. This observation suggests the presence of irreversible or weakly reversible adsorption on bare CNFs, likely due to localized trapping or limited desorption pathways. The enhanced recovery in the Au/CNF device is attributed to the catalytic role of gold nanoparticles, which facilitate surface redox reactions and promote faster electron exchange dynamics. Additionally, Au NPs help reduce potential energy barriers at the sensing interface, enabling more efficient carrier relaxation after gas removal. These results confirm that Au decoration not only boosts sensitivity but also improves response speed, reversibility, and overall sensor robustness at room temperature.

**Fig. 6:**
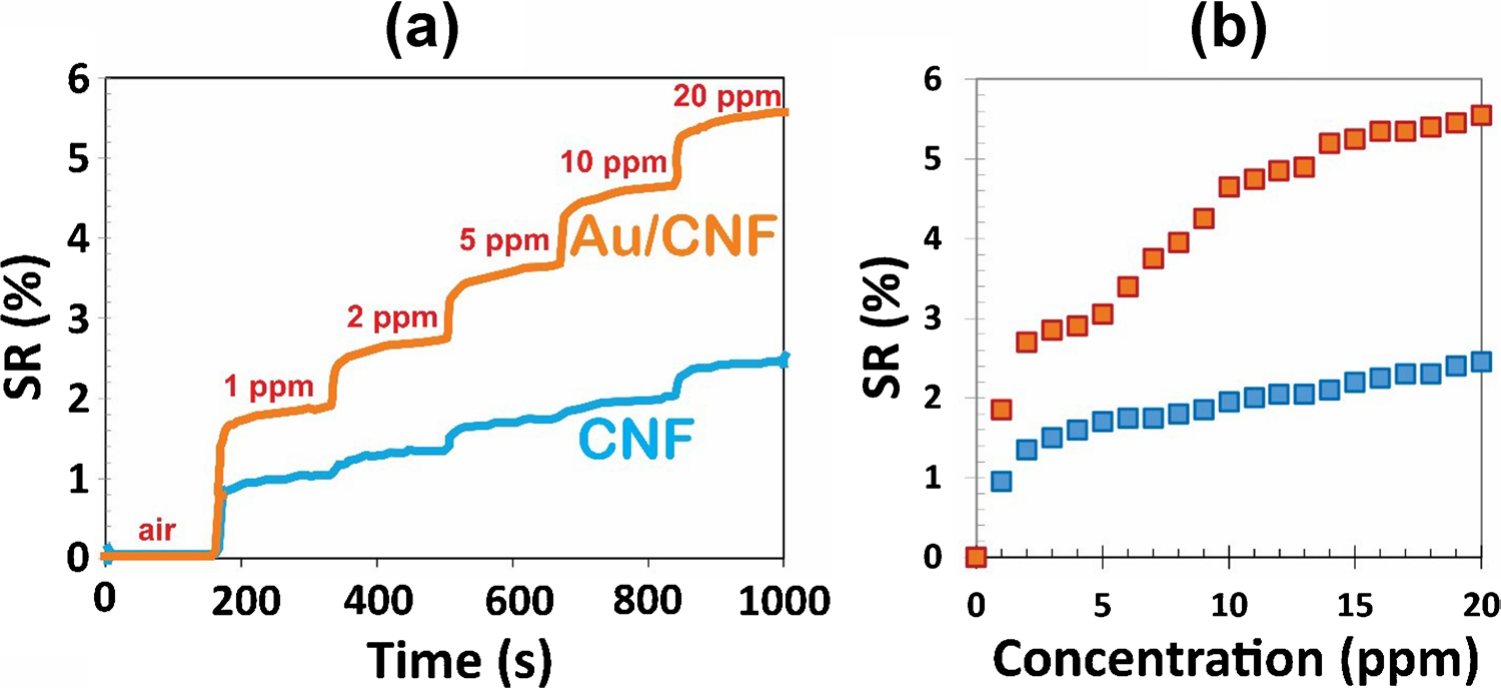
**a** Ethanol gas sensing response of pristine CNF and Au-decorated CNF (Au/CNF) sensors at various ethanol concentrations (1, 2, 5, 10, and 20 ppm) measured at room temperature. The Au/CNF sensors exhibit significantly enhanced sensitivity compared to bare CNFs due to the catalytic and electronic effects of Au nanoparticles. **b** Sensor response as a function of ethanol partial pressure, illustrating nonlinear behavior consistent with Langmuir-type adsorption, indicating saturation of available surface sites at higher concentrations

VACNFs, synthesized via DC-PECVD, exhibit intrinsic p-type semiconducting behavior, which is typical for carbon-based nanomaterials exposed to atmospheric oxygen. When a reducing gas such as ethanol is introduced, it donates electrons to the sensing layer, thereby neutralizing some of the hole carriers (majority carriers in p-type materials). This process leads to a reduction in hole concentration and consequently an increase in resistance. This behavior is consistent with previous studies on carbon-based gas sensors [46, 47]. To enhance sensitivity, Au NPs were deposited on the CNF surfaces via aerosol impaction. The addition of noble metal catalysts such as Au significantly improves the gas response through two main mechanisms: (i) spillover effect, where adsorbed gas species on Au diffuse onto the CNF surface, and (ii) electronic sensitization, where Au modifies the local electronic structure of the CNFs by forming Schottky barriers or inducing local charge transfer [48]. These effects collectively increase the number of active sites and facilitate stronger interactions between gas molecules and the sensing film. Figure 6a displays the ethanol sensing response of pristine CNFs and Au/CNFs at concentrations of 1, 2, 5, 10, and 20 ppm. The pristine CNF sensor exhibits a gradual increase in response with sensitivity values of 0.95%, 1.35%, 1.70%, 1.95%, and 2.45%, respectively. In contrast, the Au/CNF sensor demonstrates significantly enhanced sensitivity of 1.85%, 2.70%, 3.05%, 4.65%, and 5.55%, respectively, at the same concentrations. These results clearly indicate that Au NPs enhance the ethanol adsorption efficiency, likely due to increased surface catalytic activity and improved charge transfer interactions.

The response enhancement is particularly pronounced at lower gas concentrations, highlighting the role of Au in promoting surface reactivity and lowering the detection threshold. While the Au decoration enhances the sensitivity, it does not fundamentally modify the gas selectivity profile, as has been consistently reported in previous studies, where the interaction strength remains governed by the intrinsic properties of ethanol and the carbon nanofiber surface [17, 20]. Figure 6b plots the sensor response as a function of ethanol partial pressure, revealing a nonlinear, saturating trend consistent with Langmuir-type adsorption kinetics. This behavior suggests a limited number of available adsorption sites that become occupied at higher concentrations. Also, Figure S1 shows the time-resolved conductance behavior of CNF and Au/CNF sensors at various ethanol concentrations. The Au-decorated device exhibits higher baseline conductance and a stronger signal response to ethanol vapor, consistent with enhanced charge transfer and surface interaction mechanisms.

**Fig. 7:**
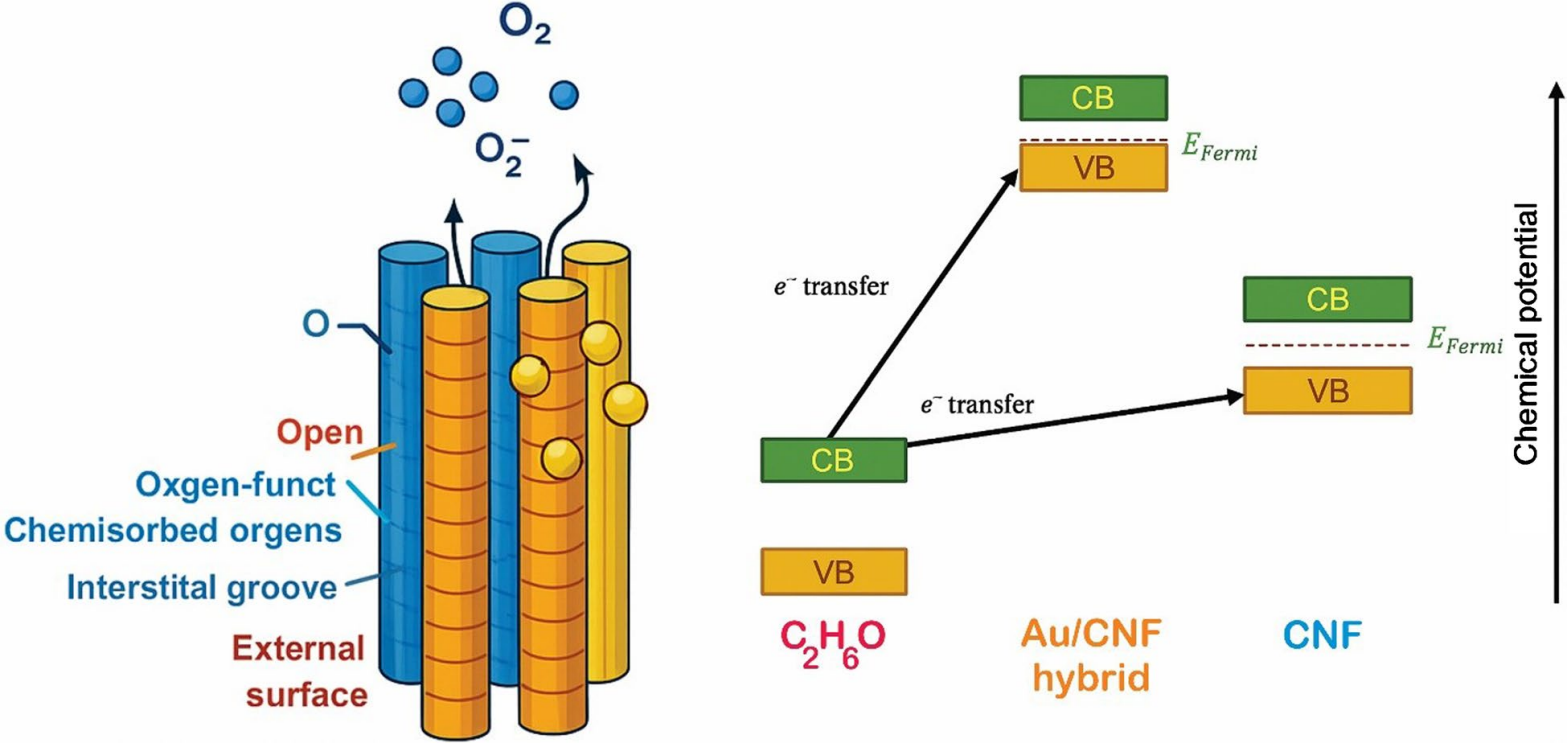
Schematic band alignment and charge transfer mechanism illustrating the sensing behavior of Au-decorated carbon nanofibers (CNFs) upon exposure to ethanol vapor. Electron donation from ethanol molecules shifts the Fermi level and modulates carrier concentration in the p-type CNF, while Au nanoparticles enhance surface chemisorption and facilitate rapid electron exchange, resulting in improved gas sensitivity

As illustrated in the schematic band diagram presented in Fig. 7, the enhanced sensing performance of the Au-decorated carbon nanofiber (Au/CNF) sensor arises from complex interfacial interactions and charge transfer dynamics. The CNFs offer multiple adsorption sites, including: (1) Au-decorated outer surfaces, (2) interstitial channels between fibers, (3) open-ended tubular pores, (4) intrinsic structural defects, and (5) oxygen-functionalized regions. These diverse sites enable efficient gas adsorption and facilitate rapid surface reactions. Gold nanoparticles (Au NPs) catalyze the adsorption of molecular oxygen (O₂), facilitating the formation of chemisorbed O₂⁻ species on the CNF surface at room temperature. These superoxide ions serve as electron acceptors, contributing to surface depletion and enabling interaction with reducing gases such as ethanol. These species capture conduction electrons from the CNFs, resulting in the formation of an electron-depleted surface layer (depletion region), thereby increasing the baseline resistance of the p-type CNFs. Upon exposure to ethanol, a reducing gas, the ethanol molecules donate electrons through surface redox reactions with the chemisorbed oxygen species. This process releases the trapped electrons back into the CNFs and reduces the hole concentration, leading to a further increase in resistance. The band diagram illustrates this mechanism through the relative positions of the valence band (VB) of ethanol and the conduction band (CB) of the Au/CNF hybrid. Electron transfer from ethanol to the Au/CNF hybrid is more efficient than to bare CNFs, owing to the modified band alignment and catalytic interface provided by the Au NPs. This electron transfer amplifies the sensor response, confirming that the Au decoration enhances sensitivity primarily through a synergistic effect involving catalytic activation, band structure modulation, and charge transfer efficiency. Consequently, the Au/CNF sensor exhibits improved sensitivity and faster response/recovery characteristics compared to the unmodified CNF sensor.

**Fig. 8:**
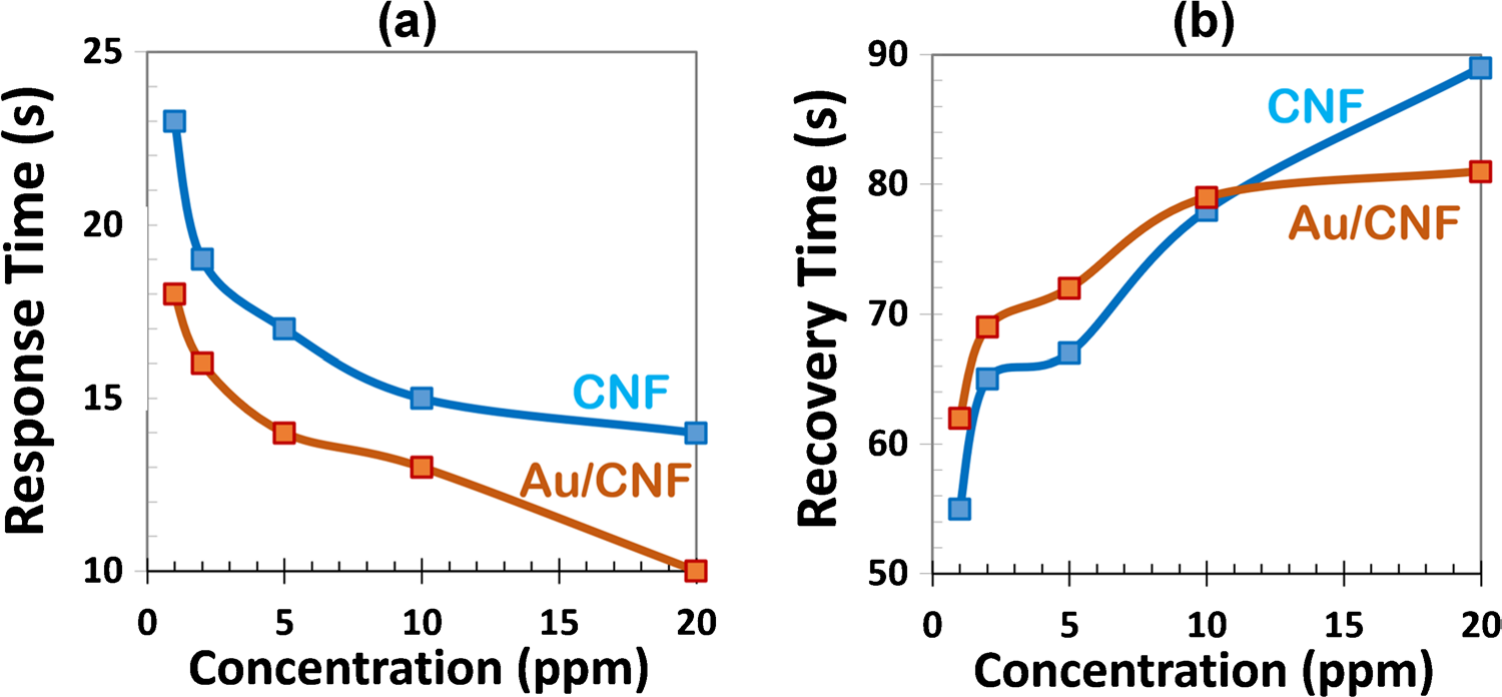
**a** Response and **b** recovery times of CNF and Au/CNF sensors at various ethanol concentrations (1–20 ppm) at room temperature

The temporal dynamics of gas sensing, quantified by the response time (time to reach 90% of the final signal upon gas exposure) and recovery time (time to return to 10% of the signal after gas removal) as presented in Figure S2, reflect the adsorption and desorption kinetics of ethanol molecules on the sensing surface [49]. As shown in Fig. 8, the response time for both CNF and Au/CNF sensors decreases with increasing ethanol concentration. This trend is consistent with the fundamental principle that higher gas concentrations introduce a greater number of analyte molecules per unit volume, increasing the collision rate with the sensor surface. At higher concentrations, the adsorption sites become occupied more rapidly, allowing the system to reach equilibrium faster and causing a quicker change in conductivity. For instance, the response time of the Au/CNF sensor decreases from 18 s at 1 ppm to just 10 s at 20 ppm, demonstrating its superior sensitivity and faster dynamics compared to the bare CNF sensor (which decreases from 23 to 14 s over the same range). The enhanced response speed of the Au/CNF sensor is primarily attributed to the presence of gold nanoparticles, which introduce additional active sites for adsorption and lower the activation energy for charge transfer. Gold facilitates the catalytic dissociation of ethanol and chemisorbed oxygen species, accelerating the initial surface reaction and promoting a more efficient and rapid modulation of charge carrier density in the p-type CNFs. This catalytic effect not only enhances the sensitivity but also shortens the response time [50].

In contrast, the recovery time shows an increasing trend with gas concentration for both sensors. This is because, at higher concentrations, the CNF surfaces become more saturated with ethanol molecules, requiring a longer time to fully desorb the gas into clean air. Desorption is often slower than adsorption, especially when the analyte forms stronger interactions with the surface (e.g., hydrogen bonding or van der Waals forces) or when it penetrates deeper into the porous or fibrous structure of the nanomaterial. For example, the recovery time for CNFs rises from 55 s at 1 ppm to 89 s at 20 ppm. A similar but slightly less pronounced increase is observed for Au/CNF sensors (62 to 81 s). Interestingly, while Au NPs facilitate faster adsorption and hence shorter response times, their effect on recovery is more complex. In some cases, the Au–ethanol interaction may be strong, especially under humid conditions, which could delay desorption. However, if the adsorption is primarily physisorption rather than chemisorption, the desorption might remain relatively fast. In my measurements, Au/CNF sensors exhibit slightly longer recovery times than CNFs at lower concentrations (e.g., 62 s vs. 55 s at 1 ppm), suggesting that Au slightly strengthens the interaction with ethanol. Yet, the difference diminishes at higher concentrations, possibly due to saturation effects [50]. In practical terms, recovery time can often be reduced by applying mild heating or forced airflow, which promotes the desorption of gas molecules and the regeneration of surface adsorption sites.

**Fig. 9:**
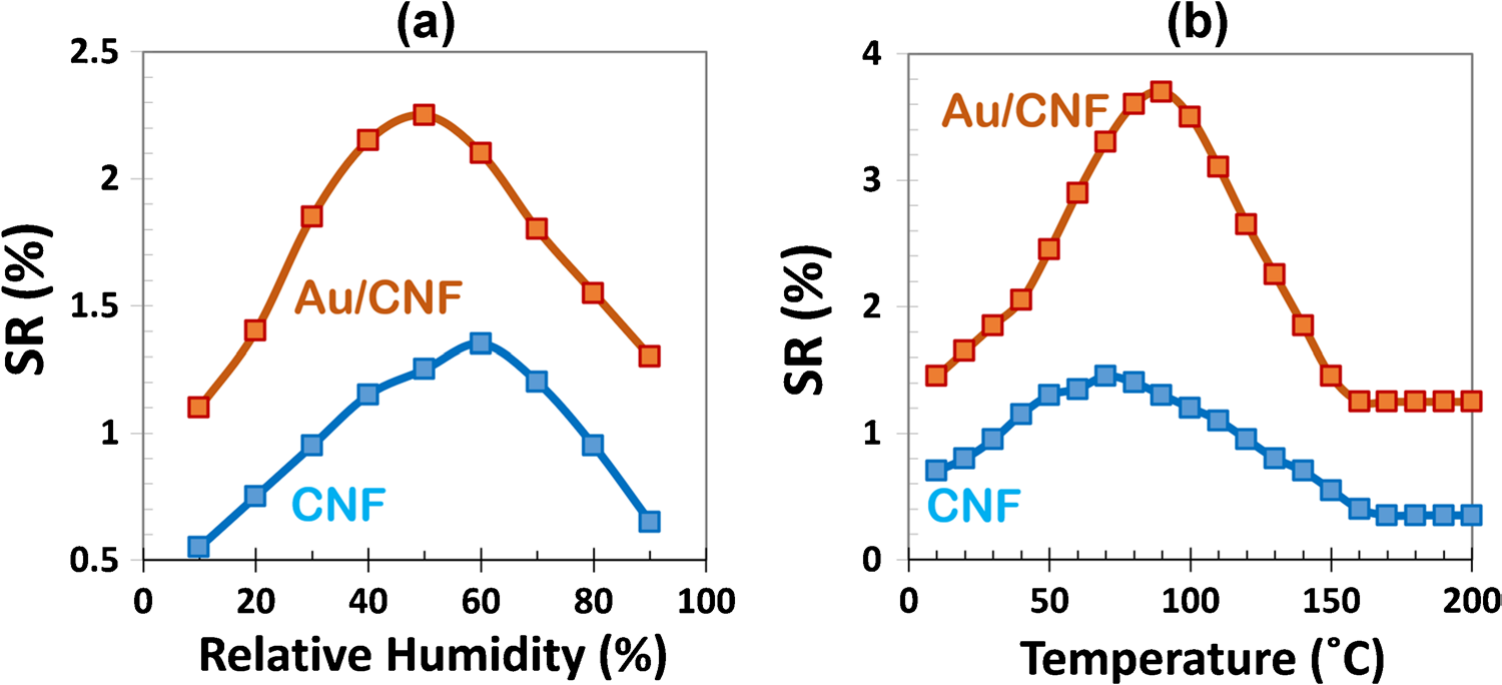
1 ppm ethanol sensing response of CNF and Au/CNF sensors **a** at various relative humidity (RH) levels at room temperature and **b** at various temperatures in RH = 30%

As illustrated in Fig. 9a, the sensing response of both CNF and Au/CNF sensors toward 1 ppm ethanol is significantly influenced by ambient relative humidity (RH). For pristine CNF sensors, the response follows a non-monotonic trend, with maximum sensitivity observed at intermediate RH levels (50–60%). This behavior can be attributed to two competing phenomena. At low RH (10–20%), the dry surface lacks sufficient adsorptive interaction sites, leading to weaker ethanol uptake and thus lower response [10]. As RH increases to moderate levels, the adsorbed water molecules form hydrogen-bonded networks that facilitate ethanol physisorption via a synergistic mechanism, enhancing the overall gas response. However, at higher RH levels (> 70%), excessive water adsorption on the CNF surface leads to competitive inhibition, where water molecules occupy active sites and hinder ethanol adsorption. Moreover, multilayer water coverage may act as a diffusion barrier, further reducing the sensitivity [33]. In contrast, the Au/CNF hybrid sensor exhibits a consistently higher and more stable response across the entire RH range. Notably, the Au/CNF sensor reaches its maximum response at a lower RH (~ 50%) compared to the CNF sensor (~ 60%), suggesting more effective ethanol interaction in less humid conditions. Moreover, the response fluctuation over the RH range is lower for Au/CNF (approximately 2 ×) than for bare CNF (approximately 2.5 ×), indicating improved signal stability and humidity tolerance due to Au decoration. This improvement stems from several advantages introduced by the Au nanoparticles. First, Au provides additional and selective adsorption sites that preferentially bind ethanol over water, due to its relatively hydrophobic nature and catalytic surface properties. Second, the hybrid system enables both physisorption and chemisorption mechanisms, enhancing ethanol interaction even in the presence of moisture [10, 49, 51]. Third, Au nanoparticles modulate the surface electronic properties of CNFs, facilitating more efficient charge transfer during gas exposure. This reduces the influence of water-induced conductivity changes and results in a more robust signal under varying humidity conditions. Therefore, the Au decoration not only improves the baseline sensitivity but also enhances the environmental stability of the sensor, maintaining high performance even at elevated RH levels.

Figure 9b presents the ethanol sensing responses of CNF and Au/CNF sensors over a range of operating temperatures (10–200 °C) under constant relative humidity (30%). Both sensors exhibit strong temperature dependence, though with distinct response profiles. The pristine CNF sensor demonstrates a typical Gaussian-like response, peaking between 60 and 70 °C. This behavior can be attributed to the thermally activated balance between adsorption and desorption of ethanol molecules on the CNF surface [10, 52]. At low temperatures (10–40 °C), although ethanol physisorption is thermodynamically favorable, the limited molecular mobility and slow kinetics of surface reactions result in weak sensitivity. As the temperature increases to the intermediate range (50–100 °C), the sensor achieves an optimal dynamic equilibrium between adsorption and desorption, leading to the highest response. Beyond 100 °C, rapid ethanol desorption dominates, reducing surface residence time and thereby diminishing sensitivity significantly. In contrast, the Au/CNF sensor shows a broader and more thermally stable response profile, with a peak sensitivity observed around 90 °C. The presence of Au nanoparticles enhances ethanol sensing performance across all temperature ranges. At lower temperatures, Au provides additional active sites and facilitates selective adsorption of ethanol, leading to a higher initial response than the bare CNF. In the intermediate regime, the synergistic effect between Au and CNF enables faster and more efficient charge transfer during ethanol adsorption, contributing to a higher and broader sensitivity peak. Notably, at elevated temperatures (> 100 °C), while the CNF sensor exhibits a sharp performance drop, the Au/CNF sensor retains a relatively strong response. This can be ascribed to the chemical adsorption of ethanol on Au surfaces and the stability of Au–ethanol interactions, which prevent abrupt desorption [11, 53]. Furthermore, the electronic modulation introduced by Au enhances the robustness of the sensing signal. These observations confirm that Au decoration not only enhances ethanol sensitivity but also extends the effective operating temperature window, improving sensor reliability and performance under thermally dynamic conditions.

**Fig. 10:**
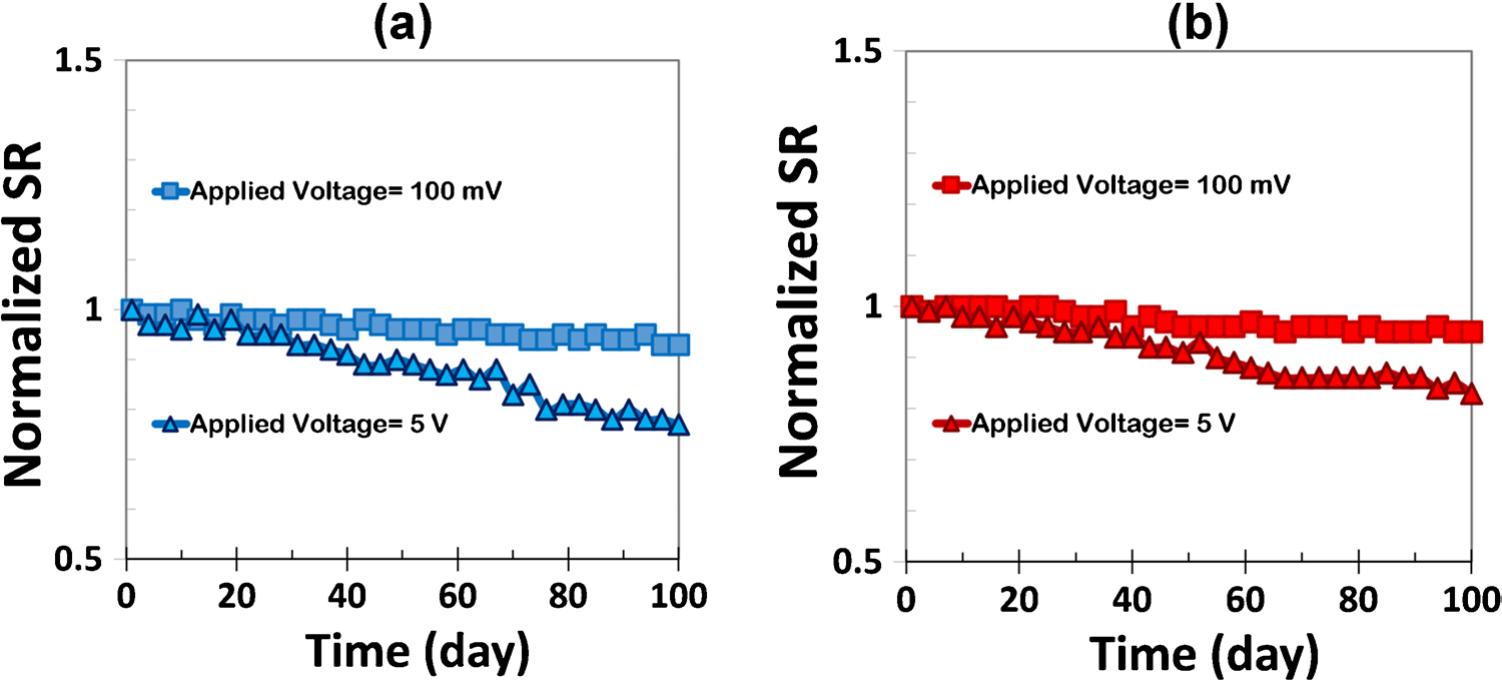
Long-term stability of CNF and Au/CNF sensors under continuous exposure to 1 ppm ethanol over 90 days at 30% RH and room temperature

To evaluate the long-term operational stability of the sensors, I monitored their response to 1 ppm ethanol over a period of 90 days at room temperature and constant RH (30%) under two biasing conditions: low voltage (100 mV) and high voltage (5 V). The results are presented in Fig. 10. Under 100 mV bias, both CNF and Au/CNF sensors maintained good stability, with only ~ 7% and ~ 5% reduction in sensitivity, respectively. This low-voltage regime is considered non-destructive, where the current remains minimal, and the structural integrity of the CNF network is largely preserved. Minor changes in response may stem from gradual adsorption of environmental contaminants, slow rearrangement of nanotube contacts, or slight variations in surface hydration. The Au/CNF sensor exhibits slightly better retention of sensitivity, attributed to the stabilizing effect of Au nanoparticles which provide catalytic and selective adsorption sites and help protect the CNF surface against environmental degradation.

In contrast, under 5 V bias, the sensors exhibit more significant degradation. The pristine CNF sensor shows a marked ~ 23% decrease in sensitivity, indicating partial deterioration of the conductive network. This is likely due to localized Joule heating at inter-tube junctions, leading to oxidation, defect generation, or even partial breakdown of covalent bonds within the nanotubes. Additionally, high electric fields may induce ionic migration from the substrate or electrodes, further contaminating or disrupting the CNF network. The Au/CNF sensor also shows a decline (~ 17%) but remains comparatively more stable than the pristine CNF, highlighting the protective and stabilizing role of Au. Gold nanoparticles contribute to enhanced long-term performance in several ways: (i) they provide additional, chemically active sites that maintain sensor response even if part of the CNF network degrades, (ii) their excellent electrical conductivity mitigates localized heating effects, and (iii) they act as a physical and chemical barrier to oxidation and environmental damage [54]. These features collectively contribute to superior electrochemical and structural resilience of the Au/CNF sensor, particularly under elevated electrical stress. In summary, both sensors demonstrate acceptable stability under low-voltage operation, but the Au-decorated CNF outperforms the pristine CNF in preserving sensing functionality over time, especially under high-voltage bias. This reinforces the potential of Au-functionalization as a strategy to enhance the durability and reliability of carbon nanomaterial-based gas sensors in real-world applications.

To benchmark the sensing performance of the Au/CNF sensor, a comparative summary with recent CNT/CNF-based ethanol sensors is provided in Table 1. As shown, the proposed device demonstrates response (~ 2% at 1 ppm), fast response/recovery times (18 s/62 s), and room-temperature operation, outperforming several reported systems that often require elevated temperatures or exhibit slower dynamics.

**Table 1:** Comparison of ethanol sensing performance between this work and selected recent CNT/CNF-based gas sensors, highlighting response magnitude, dynamic behavior, and operating temperature

Material	Ethanol Conc.	Response (%)	Response Time (s)	Recovery Time (s)	Temp	Reference
Au/CNF	1 ppm	~ 2	18	62	RT	This work
ZnO NRs/MWCNTs	100 ppm	~ 26	2	16	370 °C	[55]
Defective CNT	50 ppm	~ 8.8	90	50	30 °C	[56]
CNT-TiO_2_	5 ppm	~ 1.7	~ 50	~ 90	RT	[49]
CNTs-ZnO/PS	500 ppm	1.25	20	-	RT	[57]
ZnO-CNT	3000 ppm	2	70	25	RT	[34]

## Conclusion

In this study, I demonstrated that vertically aligned carbon nanofibers (VACNFs) synthesized via PECVD and functionalized with aerosol-printed gold nanoparticles exhibit enhanced sensitivity and selectivity toward volatile organic compounds (VOCs), particularly ethanol vapor, at room temperature. Detailed structural and compositional characterization using TEM, SEM, EDX, and Raman spectroscopy confirmed the successful integration of Au nanoparticles on the CNF surfaces and revealed key modifications in graphitic structure and surface reactivity. Notably, the presence of sub-10 nm Au NPs significantly increased the density of active sites and facilitated charge transfer interactions, as evidenced by Raman G-band upshifts and increased *I*_D_/*I*_G_ ratios. These structural modifications directly contributed to the observed improvements in gas sensing performance.

Gas sensing measurements showed that Au-decorated CNFs exhibited a markedly higher response compared to pristine CNFs under identical conditions, with a clear correlation between nanoparticle decoration and increased signal amplitude. This enhancement is attributed to the synergistic effect of the high surface area of CNFs and the catalytic, electron-donating properties of Au nanoparticles, which together promote efficient adsorption and charge modulation upon VOC exposure. Moreover, the ability to control CNF density and nanoparticle deposition with precision provides a scalable and tunable platform for sensor fabrication.

These findings not only validate the role of noble metal functionalization in advancing CNF-based sensor architectures but also underscore the potential of this approach for developing highly responsive, room-temperature gas sensors suitable for applications in environmental monitoring, industrial safety, and non-invasive diagnostics. Future work will explore long-term stability, selectivity across different VOC species, and integration into miniaturized sensing systems.

## Supplementary Information

Below is the link to the electronic supplementary material.

ESM 1 (DOCX 196KB)

## Data Availability

No datasets were generated or analysed during the current study.
