# Supplementary material for: Gold-decorated vertically aligned carbon nanofibers for high-performance room-temperature ethanol sensing
Source: Mikrochim Acta. 2025 Jul 24;192(8):517. doi: 10.1007/s00604-025-07367-8 (PMC12287182; doi:10.1007/s00604-025-07367-8)

Supporting Information:

**Gold-Decorated Vertically Aligned Carbon Nanofibers for High-Performance Room-Temperature Ethanol Sensing**

Mostafa Shooshtari^1,2*^

^1^ Neuromorphic Group, Instituto de Microelectrónica de Sevilla (IMSE-CNM), Consejo Superior de Investigaciones Científicas (CSIC) and Universidad de Sevilla, Sevilla, Spain.

^2^Department of Microelectronics, Delft University of Technology, Feldmannweg 17, 2628 CT, Delft, the Netherlands.


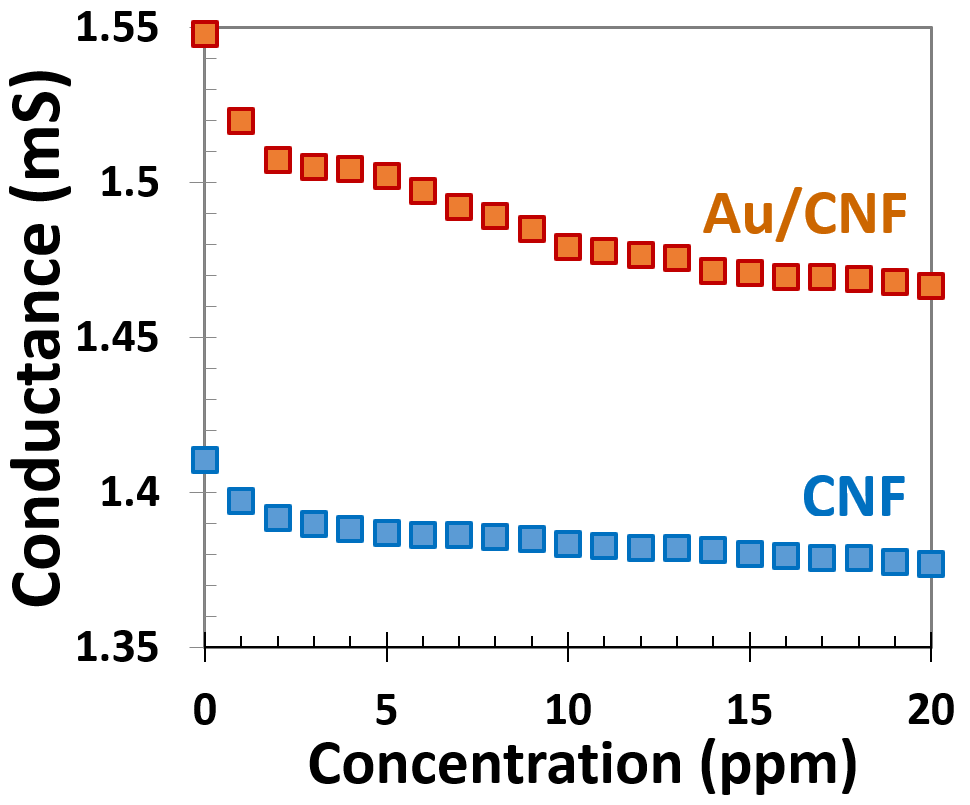


**Figure S1:** Real-time conductance response of pristine CNF and Au-decorated CNF (Au/CNF) sensors to ethanol vapor at various concentrations (1–20 ppm) at room temperature. The Au/CNF sensor exhibits a higher baseline conductance due to improved charge carrier density and reduced interfacial resistance introduced by Au nanoparticles. Upon exposure to ethanol, both sensors show a decrease in conductance (i.e., increased resistance), consistent with p-type sensing behavior. The Au/CNF sensor demonstrates a more pronounced and repeatable response, confirming enhanced gas interaction and signal robustness. (The baseline resistance values of the CNF and Au/CNF sensors were measured as **709 Ω** and **646 Ω**, respectively. All resistance measurements were performed under a constant applied voltage of **100 mV**).

| 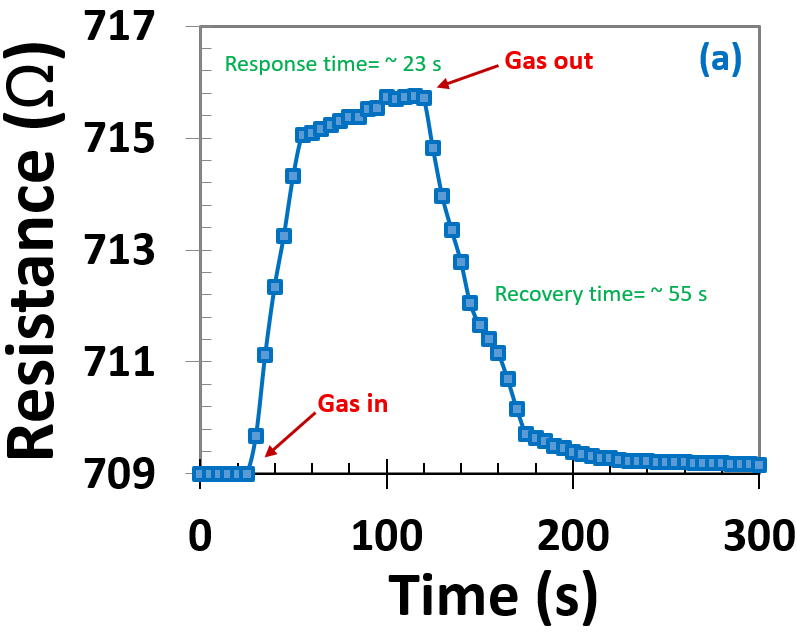 | 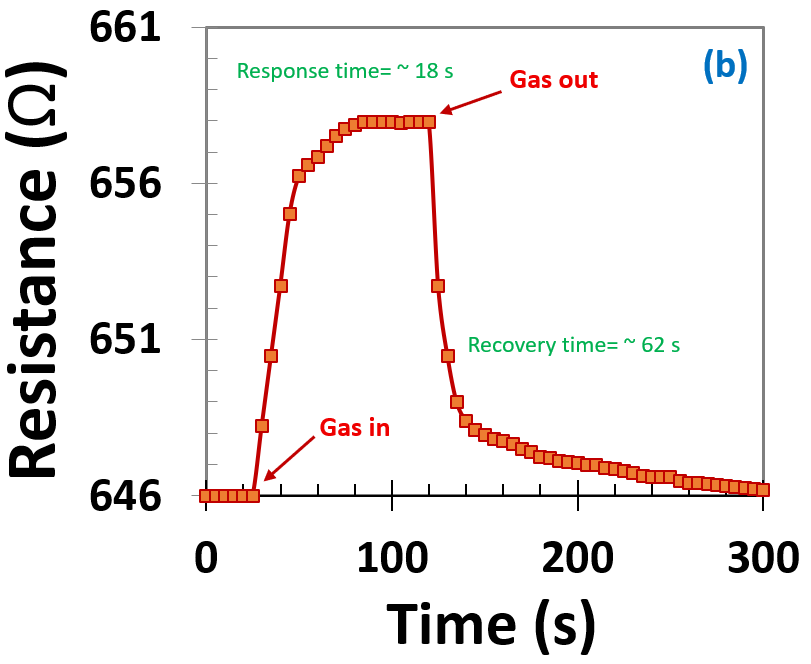 |
| --- | --- |

**Figure S2:** Dynamic resistance response curves of (a) pristine CNF and (b) Au-decorated CNF (Au/CNF) sensors upon exposure to 1 ppm ethanol at room temperature. Both plots show time-resolved resistance behavior highlighting the sensor’s response and recovery dynamics. The response time is defined as the duration required to reach ~90% of the maximum resistance change after gas exposure, while the recovery time is the time needed to return to ~90% of the baseline resistance after gas removal. The CNF sensor exhibits a response time of approximately 23 s and recovery time of 55 s, whereas the Au/CNF sensor shows a faster response time of 18 s but a slightly longer recovery time of 62 s. These results confirm that gold nanoparticle decoration enhances the ethanol adsorption kinetics and response speed, while also modulating the desorption characteristics of the sensor surface.

***Table S1:*** *Elemental composition of the Au-decorated CNF sample as determined by energy-dispersive X-ray spectroscopy (EDX). The table reports the weight and atomic percentages of each detected element, along with net intensity counts, error percentage, and correction factors (K-ratio, Z, A, F). The presence of ~16 wt% Au (~2.5 at%) confirms successful nanoparticle deposition on the CNF surface. Residual Ni corresponds to the catalyst layer used during CNF growth, and the Si signal originates from the underlying substrate*.


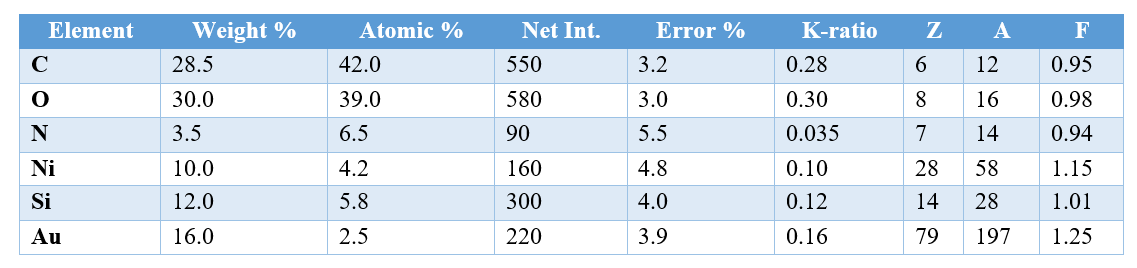

Supplement: Supplementary file 1 — (DOCX 196KB) [file 604_2025_7367_MOESM1_ESM.docx]
